# Action toward sound sources enhances auditory spatial confidence: on the metacognitive consequences of reaching to sounds

**DOI:** 10.1007/s00426-025-02079-3

**Published:** 2025-01-19

**Authors:** Chiara Valzolgher, Lisa Lever, Tommaso Rosi, Francesco Pavani

**Affiliations:** 1 Center for Mind/Brain Sciences (CIMeC), University of Trento, Rovereto, TN Italy; 2 Department of Psychology and Cognitive Sciences (DiPSCo), University of Trento, Trento, Italy; 3 Centro Interuniversitario di Ricerca “Cognizione, Linguaggio e Sordità” (CIRCLeS), Trento, Italy; 4 Level UP, Trento, Italy

## Abstract

Each perceptual process is accompanied with an evaluation regarding the reliability of what we are perceiving. The close connection between confidence in perceptual judgments and planning of actions has been documented in studies investigating visual perception. Here, we extend this investigation to auditory perception by focusing on spatial hearing, in which the interpretation of auditory cues can often present uncertainties. We asked if confidence in perceived sound position changes when we interact with the sound source by planning a sound-directed motor action (reaching) vs. a verbal response (naming). We tested 30 participants in a sound localization task in which they were both asked to localize sound sources by reaching them or by naming the labels above them in a within-participants experimental design. Participants performed the task with binaural hearing and with one ear plugged to increase errors and reduce confidence. Results showed that sound localization performance did not differ between reaching and naming, and yet participants felt more confident and required less time to complete the trial when reaching to the sources compared to naming them, regardless of the listening condition. Moreover, we found that during monaural listening the coherence between performance and confidence was reduced in each trial, irrespective of response type, suggesting increased difficulties in metacognitive monitoring. These findings suggest that, even in the case of spatial hearing, motor action planning plays a role in the formulation of confidence judgments, alongside sensory inputs and decision-making processes and stress the importance of including metacognitive measurements into spatial hearing research.

**Supplementary Information:**

The online version contains supplementary material available at 10.1007/s00426-025-02079-3.

## Introduction

Each perceptual process is accompanied with an evaluation regarding the reliability of what we are perceiving. Even when we do not explicitly ask ourselves whether we are sure of what we saw or heard, our cognitive system processes this uncertainty and uses this information actively in the perceptual process: the uncertainty of these inputs is weighted to plan actions that have higher probability of success (Fleming, 2021; Giesel et al., 2020; Hohwy, 2013). One context in which perception is uncertain is when we attempt to determine the direction of sounds. It is a common experience to hear sounds whose position is uncertain, such as when we wander around our apartment searching for our ringing smartphone without visual cues, or when we hear hisses that we cannot easily locate. Yet, research on spatial hearing has largely neglected perceived uncertainty in sound localization, and has not explored the interactions between goal-directed motor behaviour and confidence in the domain of sound localization.

The connection between confidence in perceptual judgments and planning of actions has been documented in laboratory studies. Confidence about a certain sensory perception is, in part, processed after the action implementation of the response about it. Siedlecka and colleagues (2021) instructed participants to manually respond, implementing a motor action, if they answered affirmatively to a question displayed on the screen, and not to respond if they answered negatively. After each trial, they were also asked to estimate their own confidence in their choice or to report an error in their decision. The confidence ratings were higher in trials in which the response was provided through an action. Other studies adopting neuroimaging and neurostimulation approaches are in line with these results. Fleming and colleagues (2015) demonstrated that disturbing the circuits involved in action processing determines an alteration in the feeling of confidence. They used transcranial magnetic stimulation (TMS) on the premotor cortex during a visual perceptual judgment task that caused a modification of the confidence judgment expressed on their perceptual discrimination. This suggested that the premotor cortex activity interacts with the accuracy of the confidence decision. Along the same line, recent studies investigated this relationship in visual perceptual tasks by using electroencephalography recordings (Sanchez et al., 2023; Wokke et al., 2020). Taken together, these results indicate that motor action plays a role in the decision process and thus in the estimation of subjective confidence about the response implemented on the sensory perception.

Despite the fundamental role uncertainty estimation plays in everyday actions and decision making, many studies on spatial hearing abilities have often omitted to collect participants' feelings of confidence regarding their perceptions. Some studies in this research field examined uncertainty by considering the distribution of participants response (i.e. precision). In this perspective, higher precision could reflect higher level of confidence, while lower precision (higher variability in the response distribution) could be an index of lower confidence (e.g., Garcia et al., 2017). However, only a few pioneering studies were interested in the feeling of confidence accompanied with the perception of sound position (Rabini et al. 2020; Valzolgher et al., 2024). These studies measured participants’ performance and confidence in altered listening conditions (e.g., simulated monaural listening or listening with background noise).

Specifically, Rabini and collaborators (2020) measured performance and the related feelings of confidence in a group of normal hearing adults who listened in normal and altered listening conditions (i.e., simulated monaural listening obtained by plugging one ear). Participants were exposed to 500 ms Italian syllables spoken by a female speaker, and then asked to choose the position of the speaker. For doing so, they should select a region of a panel composed by 60 speakers positioned in front of them (no visual cues about the speakers were provided). After indicating sound position, they were asked to provide a subjective rating of their confidence about the perceived sound position. The authors observed that monaural listening not only altered performance but also leads to a decline in the perceived confidence. In addition, the authors observed that accuracy and feeling of certainty dissociated when they listen with one ear plugged, meaning that in some trials participants were certain but incorrect, while in others trials they were uncertain but correct. Valzolgher et al. (2024) tested normal-hearing participants in a speech-localization task while they were immersed in three common soundscapes that progressively increased in complexity: nature, traffic, and a cocktail party setting. They found that not only performance decreased, but also the feeling of confidence reported by participants decreased as the complexity of the background noise increased. So, we can conclude that even with an ear plug or in adverse environments, people might still be able to approximately identify the position of sound sources, but they might be much more uncertain that the position indicated is the correct one.

While some preliminary works have examined confidence in sound localization, it remains unclear if this can be influenced by acting on the sound source. Many of our actions and interactions are toward objects that make sounds, and yet audible objects can evoke actions. For example, hunting animals can run toward their prey, triggered by the sound of its presence; or we quickly reach out to grab our mobile phone, triggered by its ringtone. Unlike vision, where the spatial information about visual objects has – to some extent – a direct mapping onto the retina, no such direct mapping for sound position exists at the level of the receptors’ surface on the cochlea. Instead, spatial hearing results from the interpretation of binaural and monaural auditory cues, with an intrinsic greater degree of spatial uncertainty compared to vision. Yet, for the purpose of the present study, it is worth noting that one of the documented spatial maps of sound position in the brain resides in the superior colliculus. This structure receives input from hearing, vision and touch sensory modality and is pivotal in the transformation of these multisensory inputs into orienting motor actions in the external environment (King, 2004; Thevarajah et al., 2010).

Given the link between spatial hearing and motor action planning, we asked whether the confidence in one’s response on the sound’s position changes if we interact with it by planning a motor action or not. The behavioural study of Siedlecka and colleagues (2021) sought to answer this question in a visual context using a go-no go paradigm in which people were asked, in a perceptual task, to act to respond or not to act. Differently, in our study, we decided to test participants in two different conditions in which we specifically manipulated the presence or absence of a motor action directed toward the space of the sound in the response phase. We asked participants to respond by reaching the sound source (which involves planning and executing a motor action toward the space occupied by sound sources) or by naming the label positioned above the source (which did not require to implement spatially-oriented action). This comparison has been adopted in previous studies focusing on sound localization relearning mechanisms in both normal hearing people in altered listening condition (Valzolgher et al., 2020a, 2023a) and in people with hearing deficits (Alzaher et al., 2023; Valzolgher et al., 2022, 2023b).

Specifically, we asked participants to localize sounds sources by reaching toward them in a virtual reality setting by moving a controller they held in their hands and “touching” the target choosing one from an array of 13 visible speakers in front of them in two different listening conditions (i.e. binaural and monaural, meaning with one ear plugged). As a control condition, we instructed the same participants to localize sound sources by naming the label positioned above them. In all conditions, participants were then asked to rate their confidence level regarding their performance after providing their response. In order to avoid adjusting both performance and confidence judgments, we did not provide explicit feedback about the correctness of their response. Moreover, we decided not to let participants move their head freely as these movements could provide implicit information about correctness of their response (see Wallach 1940). Consistent with the studies presented above (Siedlecka et al., 2021; Wooke et al., 2020; Sanchez et al., 2023), we expected that implementing motor action toward sound sources increase confidence in one's response as compared to naming a label. Note that, although the act of naming also involves the implementation of motor movements of the organs involved in producing speech, our prediction is based on the fact that the motor action implemented in the act of reaching occurs in external space and consists of orienting an effector toward a visible external target, as was the case in the cited studies.

## Materials and methods

### Participants

We recruited 30 normal-hearing participants among the students of the University of Trento. We excluded one participant (ID = 2) because of an error in the experimental procedure and one participant because of lack of data after filtering (ID = 22, see 2.4. Data Analsyis). Mean age for the remaining participants was 22,7 years (SD = 6,9; 19 females, 25 right-handed). Handedness was determined using the short version of the Edinburgh headedness inventory (Oldfield 1971; Veale 2014). All participants had normal or corrected-to-normal vision and normal hearing (average pure tone audiometry threshold across frequencies 6.71 ± 3.12 dB HL). Hearing thresholds were measured using an audiometer (Grason Stadler GSI 17 Audiometer), testing different sound frequencies (250, 500, 1000, 2000, 4000 Hz) on the right and left ears, separately. The study was conducted in line with the Declaration of Helsinki (2013) and according to the research ethics regulations of the University of Trento (protocol: 2022–009). Participants read and signed informed consent before taking part in the experiment and received a certificate of participation to obtain educational credits or a gadget.

To the best of our knowledge, no previous studies has investigated the difference between reaching and naming responses on perceived confidence when interacting with sound sources. Thus, we determined sample size based on the documented effect of different listening conditions (binaural vs. monaural) on confidence, based on Rabini and colleagues (2020, Exp. 2). Using a within-subject experimental design, they observed an effect size of 1.22 when comparing confidence between binaural and monaural conditions (N = 20). With a power of 0.95, we calculated that only eleven participants were needed to detect this effect. Because we did not expect an effect size comparable to the contrast between monaural vs. binaural listening with our manipulation (reaching vs. naming), we adapted our power calculations to a more modest effect size of 0.5. Consequently, with a power of 0.75, we opted to test 30 participants. It is worth noting that previous studies comparing reaching and naming in terms of performance used a between-experimental design and included a total of 28 participants, split into two groups of 14 (Valzolgher et al. 2020a).

### Apparatus

**Fig. 1:**
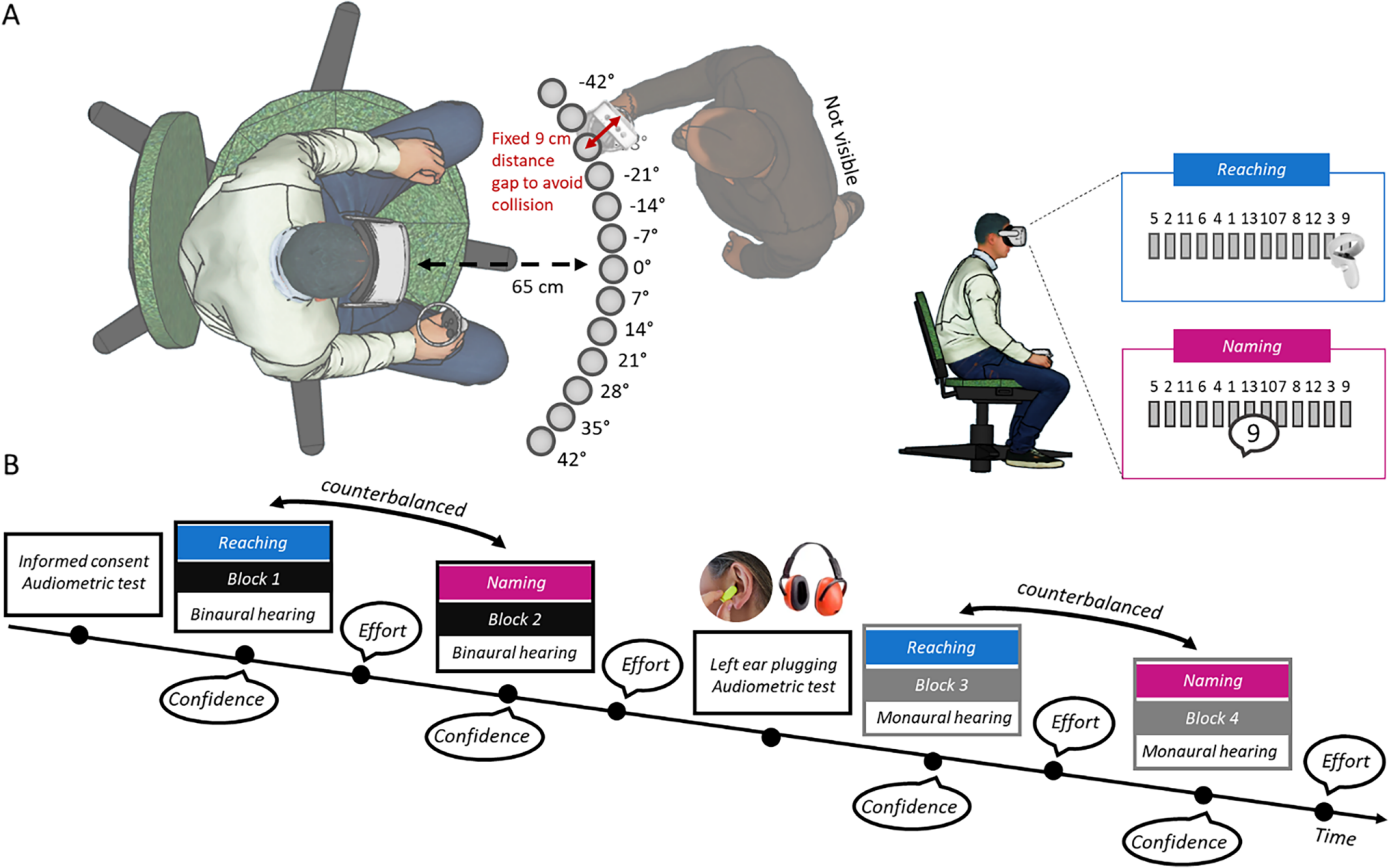
**A Setting**: schematic representation of the participant wearing the head-mounted display (HMD) and holding the virtual reality (VR) controller during the sound-localization task. The 13 cylinders in front of the participant indicate possible speakers’ positions and they are visible in the HMD. In the blue box to the right, a schematic representation of the reaching type of interaction in which participants were instructed to move the controller toward the speaker emitting sound. In the purple box to the right, a schematic representation of the naming type of interaction in which participants were instructed to name the label above the speaker emitting sound. **B** *Experimental* procedure: after informed consent and audiometric test, participants took part in 4 consecutive blocks (51 trials each) in two listening conditions (binaural in black and monaural in grey). During each trial, participants listened to white noise and were asked to identify the speaker emitting sound. At the end of each trial, they were asked to evaluate their confidence using a Likert scale. At the end of each block, they were asked to evaluate their perceived effort using a Likert scale. Note that before starting the monaural condition, we plugged one ear and performed another audiometric test on the plugged left ear. All participants performed the task in both listening condition (binaural and monaural) and in both type of interaction (reaching, in blue and naming, in purple). The order of condition was counterbalanced

The experiment was conducted in a soundproof and partially anechoic booth (Amplifon G2 × 2.5; floor area = 200 X 250 cm, height = 220 cm). Visual virtual reality (VR) and kinematic tracking was implemented using a Head-Mounted Display (HMD; Meta Quest 2; 256 GB; resolution: 3616 × 1840; frequency: 72 Hz) and 2 controllers (one was used by participants to respond, and the other was used to track the speaker's position in real-time). All stimuli were controlled and delivered using a computer (ASUS TUF Dash F15) connected to the HMD via an Oculus link cable and using a custom-made software developed in Unity (Unity Technologies) (see Fig. 1A).

After wearing the HMD, participants found themselves immersed in an empty virtual room, which mimicked the real room’s dimensions. In front of the participants, 13 visible speakers were positioned at ear level. Auditory stimulation was controlled by the Unity software and delivered using a loudspeaker emitting white noise amplitude modulated at 2.5 Hz (Valzolgher et al. 2020a; Valzolgher et al. 2020b) at about 65 dB SPL as measured by a phonometer place at ear level. The speaker was placed manually by the experimenter in pre-determined spatial positions (we used only one portable speaker to deliver the target sounds). Note that the positions were determined in real-time and on a trial-by-trial basis, always considering the position of the participant’s head. The experimenter placed the speaker according to visual instructions on a computer monitor (see Valzolgher et al., 2024 for details about this sound delivery method and Gaveu et al., 2022 for details about the original method inspiring our new technology). Note that the software delivered the target sound only when the loudspeaker reached the target position for the trial and the participant’s head was facing straight ahead.

The speaker was placed in one of 13 possible positions, which changed randomly across trials. The positions were at ear level, 65 cm from the head, and in one of 13 different horizontal positions (0°, ± 7°, ± 14°, ± 21°, ± 28°, ± 35°, ± 42°, with respect to the participant’s midsagittal line) (see Fig. 1A). The rationale to choose a small range of space (84°) in which to place the speakers comes from the fact that we wanted all the speakers to be visible even without making head movements. All the target virtual speakers were clearly visible in front of the participants. They appeared as cylinders floating in the air. The reason we used visual virtual reality combined with this particular technology to present real sounds is related to the possibility of creating a reaching condition in which participants could interact with the sounds by touching and stopping them, as well as the need to measure and control head movements.

### Procedure

Before starting the experiment, participants signed the informed consent and their hearing threshold was measured. Next, participants sat on a rotating, armless chair with no chin rest; were asked to wear the HMD and were instructed on the procedure of the experimental task.

During the sound localization task participants completed 208 trials divided into 4 blocks (Fig. 1B). The first two blocks were always completed in binaural listening condition, while the second two block in monaural listening condition. To simulate monaural hearing, in the middle of the experiment we plugged the left ear of each participant by using one ear plug (3 M PP 01 002; attenuation value for high frequencies = 30 dB SPL; attenuation value for medium frequencies = 24 dB SPL; attenuation value for low frequencies = 22 dB SPL; Single Number Rating = 32 dB SPL; as reported by the manufacturer). In addition, a unilateral earmuff covered the left ear (3 M 1445 modified to cover only the left ear; attenuation value for high frequencies = 32 dB SPL; attenuation value for medium frequencies = 29 dB SPL; attenuation value for low frequencies = 23 dB SPL; Single Number Rating = 28 dB SPL; as reported by the manufacture). Before wearing the muff, we performed again the audiometry to the left ear to measure the effect of ear plug (average threshold left ear with plug 36.36 ± 3.73 dB HL; average difference between the threshold of left ear with and without plug: 29.90 ± 4.00 dB HL). We plugged the left ear in all participants, because prior works found no difference in performance based on plugged ear side (Rabini et al., 2020).

Both binaural and monaural blocks were performed adopting two different response types, consisting in two different ways to interact with sound sources: reaching to and naming them. The order of these response conditions was randomized and counterbalanced between participants (half of the participants completed the task with the reaching-naming-reaching-naming sequence, and the remaining half with the naming-reaching-naming-reaching sequence).

At the beginning of the trial, participants were asked to direct their heads to the centre of the speakers’ array. A cross indicating their straight-ahead position was presented and it moved with their head movements to help them identify their head orientation in the virtual environment. The cross turned from red to green when placed in the correct position, signalling participants that they achieved straight ahead posture. At sound onset, the cross disappears and participants were instructed to refrain from moving their heads and trunks. Small adjustment movements were permitted only during the response phase to favour natural behaviour during the action. After presenting the auditory stimulation, participants were asked to identify the position of the sound source. There was no time restriction and the sound lasted until they provided a response.

In the reaching to sounds condition, they were instructed to move the controller in their right hand to the perceived location of the sound, as to collide the speaker (Fig. 1A). As soon as the virtual controller touched the virtual speaker, the hand-held controller vibrated to mark the collision and the sound stopped. Note that the real speaker moved by the experimenter was positioned 9 cm further back with respect the virtual position to avoid direct collision between the participant and the speaker in the reaching condition. In the naming condition, they were asked to respond by reading aloud the number located above the source speaker. The label changed randomly on a trial-by-trial basis. With the purpose to make the two response conditions visually similar, labels appeared above the speaker also in the reaching task. As in the reaching condition, when participants named the label, the sound stopped and the controller they held in their hand vibrated. In the naming condition the sound was stopped by the experimenter by pressing a button on the controller she held in her hand. This approach allowed participants to feel as though they were halting the sound by verbalizing the label of the speaker (see Supplementary Videos at osf.io/rsvhp).

In both conditions, after validating the response, participants were asked to judge their confidence about perceived sound position on a continuous scale *(“How sure you are to have correctly localized the sound?”* from 1 = not sure to 6 = sure; the original version: “Quanto sei sicuro/a di aver localizzato correttamente il suono?”). At the end of each block, they were asked to evaluate the effort perceived during the block on a continuous scale *(“How much effort did you do to localize the sounds in this part of experiment?”* from 1 = none to 6 = much; the original version: “Quanto sforzo hai fatto per localizzare i suoni in questa parte di esperimento?”). Those questions appeared on the wall of the virtual room behind and above the speakers, with the numerical scale visible. The participants responded verbally and the experimenter select the response manually. The experimenter read out loud the question and remember the scale of the response to the participant, if needed. The entire experimental session lasted about 1 h and 30 min. At the end of the session, the experimenter debriefed participants about the study and its purpose.

### Data analysis

*Pre-processing*. We started the pre-processing by considering 29 participants. 0.6% of trials were removed from analyses due to errors in sound delivery (e.g., the experimenter moved the speaker during sound emission or stopped the sound before the response). In addition, because participants were instructed to refrain from head movements (note that they were permitted to move their heads only during the response phase, to read the label or to accompany the hand movement), we examined each trial to remove trials in which participants did not comply with this instruction. To remove trials in which such movements prevailed, we identified and eliminated all the trials in which the participants' head direction fell outside 5 degrees from straight-ahead fixation, either vertically or horizontally, for more than 50% of trial time. We applied this cut-off to all the trials whose sound duration was longer than 2 s. This filtering procedure led to the removal of 15% of the trials. With this filter participant 22 obtained a significant reduction of trials, with only 3 trials on 52 left in the binaural naming condition and 4 trials on 52 on the binaural reaching condition. Consequently, we decided to exclude this participant from the analysis. Therefore, a total of 28 participants were included in the study. Data filtering was implemented directly in Unity.

To study performance, we measured the absolute and the signed error along the horizontal dimension. These indices were obtained by calculating the discrepancy between the position of the speaker and the participant response for each trial: when the participant’s response was to the right of the target, the signed error was positive. Conversely, when the participant’s response was to the left of the target, the signed error was negative. The absolute error refers to the magnitude of the error, regardless of its sign, and is calculated as the absolute value of the signed error. To study confidence and effort, we used participants’ rating as dependent variables. In addition, we considered the sound duration as a dependent variable. It is important to note that participants were not under any time restrictions and were free to let the sound play until they felt ready to respond. Therefore, we hypothesized that the sound duration could serve as an implicit index of internal uncertainty, as participants who require more time to complete the task may be struggling and undecided when providing their answer (Visentin et al., 2021).

We also computed a measure intended to capture the relationship between absolute error, serving as an index of performance, and confidence. This measure represents the consistency between performance and confidence assessments, thereby indicating participants' ability to monitor their performance in terms of internal feelings, which reflects a broader skill known as metacognition (Fleming, 2024; Fleming & Lau, 2014; Lai, 2011; Thomas et al., 2022). To compute this measure, we initially determined the disparity (i.e., the arithmetical difference) between the absolute error and the reverse of the confidence rating (i.e., 7—confidence rating). The rationale behind this calculation was that if a participant maintains a consistent internal criterion for evaluating their performance, this difference should remain constant regardless of its numerical value. Then, we proceeded to calculate the standard deviation of this distribution, as it represents the dispersion of this measure. From now on, we refer to this computed variable as "internal variability index": higher variability signifies lower consistency of the internal criterion, reflecting lower abilities to adapt one's own feelings of confidence to the expressed performance (lower metacognitive abilities). Conversely, lower variability indicates higher consistency and thus better metacognitive abilities.

Analyses were carried out using JASP 0.18.3.0 and plots were generated by using R (version 4.4.1; library ggplot2) and R-studio environment. Data can be retrieved from osf.io/rsvhp.

## Results

### Reaching vs. naming sounds in binaural and monaural listening

**Fig. 2:**
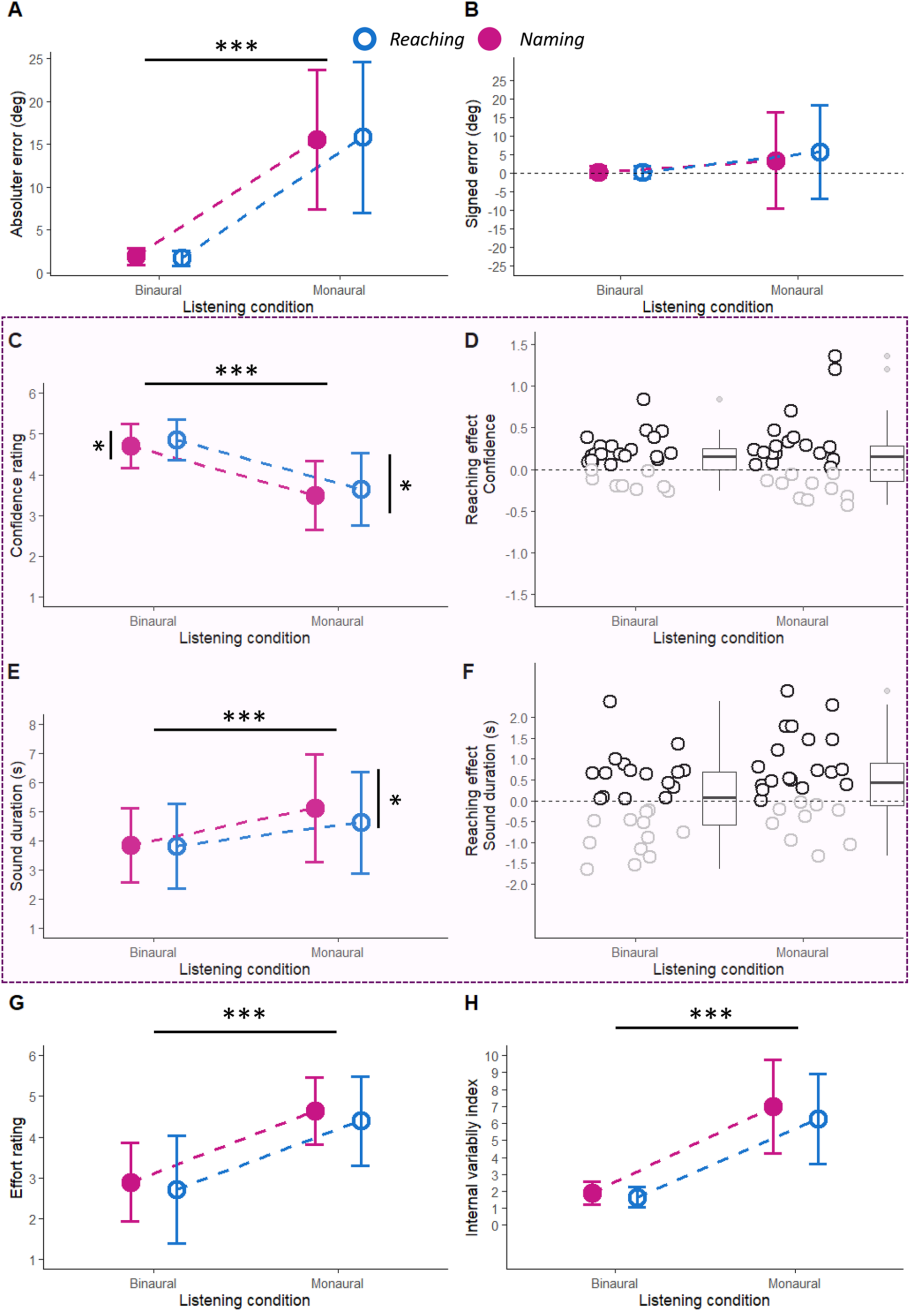
*Performance* Absolute error (**A**) and signed error (**B**) as a function of listening condition (binaural, monaural) and response type (reaching in blue, empty circles and naming in purple, full circles). The error bars represent the standard deviation. *Confidence* (violet square) Confidence ratings (**C**) and sound duration (**E**) as a function of listening condition and response type. Delta of confidence rating (i.e., the difference between the confidence ratings in reaching and in naming conditions) (**D**) calculated for each participant and showed as a function of listening condition. Most of them (in black) have a positive value, meaning that confidence ratings are higher in the reaching condition. Delta of sound duration (i.e., the difference between the confidence ratings in naming and in reaching conditions) (**F**) calculated for each participant and shown as a function of listening condition. Only in the monaural condition, most of them (black) have a longer sound duration in the naming condition. *Effort* rating (**G**) as a function of listening condition and response type. *Internal* *variability* index (**H**) as a function of listening condition and response type

*Performance.* To examine performance changes as a function of listening condition and response type, we entered absolute error and signed error in two separated analyses of variance (ANOVA), with listening condition (binaural, monaural) and response type (reaching, naming) as within-subject independent variables. For absolute error, we found a main effect of listening condition (*F*(1,27) = 77.17, *p* < 0.001, *ŋ*^*2*^ = 0.72) revealing that absolute error increased in monaural (15.67° ± 8.25°) compared to binaural listening (1.80° ± 0.82°) (Fig. 2A). No other main effect or interactions were observed (all *ps* > 0.48). For signed error, we found a main effect of response type (*F*(1,27) = 4.36, *p* = 0.046, *ŋ*^*2*^ = 0.006) and the two-way interaction (*F*(1,27) = 6.35, *p* = 0.02, *ŋ*^*2*^ = 0.008). Signed error was more positive (i.e. indicating a rightward bias) during reaching compared to naming, but only in monaural listening (*t* = 3.24, *p* = 0.01; binaural: *t* = 0.19, *p* = 1.00, Bonferroni corrected) (Fig. 2B). This result reflects the well-documented lateral shift of responses opposite the plugged side, here the left ear (Valzolgher et al., 2020a; Strelnikov et al. 2011; Mendonça, 2014; Gougoux et al., 2005). In addition, this bias likely became more evident during the reaching response due the right-hand use (Stins et al., 2001).

*Confidence.* To examine changes in confidence as a function of listening condition and response type, we entered confidence ratings in an ANOVA with listening condition and response type as within-subject variables. We found a main effect of listening condition (*F*(1,27) = 89.19, *p* < 0.001, *ŋ*^*2*^ = 0.72) revealing a decrease in confidence during monaural (3.56 ± 0.84) as compared to binaural (4.78 ± 0.50) listening (Fig. 2C and D). Interestingly, we also found a main effect of response type (*F*(1,27) = 7.70, *p* = 0.01, *ŋ*^*2*^ = 0.01), with no 2-way interaction (*p* = 0.07). This result suggests that, despite the numerical trend increase in rightward bias in performance observed specifically for the reaching condition, participants' overall confidence was higher when interacting with sound sources by reaching them (4.23 ± 0.62), as compared to naming their labels (4.09 ± 0.63). Figure 2D shows the difference in confidence ratings between the monaural and binaural listening condition for each participant. Note that most of the circles have a positive value (drawn in black for clarity), meaning that confidence expressed by participants was higher in the reaching condition than in the naming condition.

To further examine internal confidence in sound localization, we also examined sound duration. Because participants were allowed to listen to the sound as long as they wished before providing their response, longer sound durations could indicate a greater amount of time needed to decide which source to select, thus serving as indirect measure of confidence. The ANOVA on sound duration revealed a main effect of listening condition (*F*(1,27) = 31.05, *p* < 0.001, *ŋ*^*2*^ = 0.36), caused by longer sound duration in monaural (4.88 s ± 1.73 s) as compared to binaural (3.83 s ± 1.28 s) listening (Fig. 2E and F). Moreover, a significant interaction between response type and listening condition (*F*(1,27) = 5.16, *p* = 0.03, *ŋ*^*2*^ = 0.02) also emerged (main effect of response type: *F*(1,27) = 3.00, *p* = 0.09, *ŋ*^*2*^ = 0.02). These results revelated that sound duration was longer during naming vs. reaching condition in monaural listening (*t* = 2.72, *p* = 0.05), but not in binaural listening (*t* = 0.12, *p* = 1.00). In other words, during monaural condition, participants required more time to select the speaker when they named the speaker labels (4.48 s ± 1.49 s), as compared to when they reached for them (4.22 s ± 1.49 s). Figure 2F shows the difference in sound duration between the monaural and binaural listening condition for each participant. Note that most circles have a positive value (drawn in black for clarity), meaning that response times were higher in the naming condition than in the reaching condition, but only in the monaural listening condition.

Since this difference in sound duration was not observed during binaural listening, we attribute this effect to confidence-related decision processes rather than experiment-related procedural differences (recall that, during the naming condition, the experimenter had to manually stop the sounds, while this was an automatic procedure during reaching, see Methods). Further evidence in support of this interpretation comes from the study of the correlation between the confidence cost of changing the listening condition from monaural to binaural (computed as the difference in confidence between listening conditions, for each participant) and the sound duration cost for the same change in listening condition (computed as the difference in sound duration between listening conditions, for each participant). When studied separately for reaching and naming, using Pearson correlation, we found a significant relationship for both reaching (*R* = 0.40, *p* = 0.04) and naming (*R* = 0.51, *p* = 0.005) (see Supplementary Fig. 1). The higher the confidence cost (where positive values indicate a greater decrease in confidence when changing from the binaural to monaural listening), the higher the sound duration cost (where positive values indicate a greater increase in the amount of time in seconds when changing from the binaural to monaural listening). These results corroborate the idea that time duration could be interpreted as an implicit measure of confidence experienced while localizing sound sources (Kiani et al., 2014).

*Effort rating*. A similar ANOVA on effort rating provided by participants at the end of each block revealed a main effect of listening condition (*F*(1,27) = 118.72, *p* < 0.001, *ŋ*^*2*^ = 0.67) indicating higher effort rating duration during the monaural (4.48 ± 0.89) as compared to binaural (2.79 ± 1.05) listening condition (Fig. 2G). The main effect of response type also approached significant (*F*(1,27) = 2.73, *p* = 0.11, *ŋ*^*2*^ = 0.011) with a numerical trend for higher perceived effort during naming (3.76 ± 0.77) as compared to reaching condition (3.52 ± 1.09). This result, although not statistically significant, aligns with previous observations regarding confidence and sound duration (Table 1).

**Table 1:** Mean and standard deviation of the recorded variables (absolute error, signed error, confidence rating, sound duration, effort rating, internal consistenty index) as a function of listening condition (binaural and monaural) and response type (reaching and naming)

Listening condition	Binaural		Monaural	
Response type	Reaching	Naming	Reaching	Naming
Absolute error (deg)	1.67 ± 0.88	1.90 ± 0.97	15.80 ± 8.80	15.54 ± 8.13
Signed error (deg)	0.19 ± 1.62	0.32 ± 1.58	5.65 ± 12.65	3.43 ± 12.94
Confidence rating	4.85 ± 0.50	4.71 ± 0.54	3.64 ± 0.89	3.48 ± 0.84
Sound duration (s)	3.82 ± 1.45	3.84 ± 1.27	4.46 ± 1.85	5.12 ± 1.85
Effort rating	2.71 ± 1.32	2.89 ± 0.96	4.39 ± 1.10	4.64 ± 0.83
Internal variability index	1.65 ± 0.61	1.91 ± 0.69	6.27 ± 2.66	6.99 ± 2.76

### The internal variability index

Having measured performance and confidence in each single trial, we examined the coherence in the trial-by-trial alignment between performance and confidence as a function of listening condition and response type. As detailed above (data analysis), we computed for this purpose an internal variability index, which entered in an ANOVA with listening condition (binaural, monaural) and response type (reaching, naming) as within-subject independent variables. This analysis revealed a main effect of listening condition (*F*(1,27) = 119.06, *p* < 0.001, *ŋ2* = 0.69) due to a lower variability during the binaural (1.78 ± 0.51) as compared to monaural listening condition (6.63 ± 2.20) (Fig. 2H). No other main effect or interaction reached significance (ps > 0.13). This result suggests that during binaural hearing, participants were able to maintain a more consistent criterion between their performance and the level of reported confidence. However, during the monaural listening condition, this criterion appeared less stable, likely because this type of listening experience was completely new for them.

### Complementary analyses

We further inspected the data by correlating a measure of the plug effect, calculated as the difference between the auditory threshold measured in the left ear with and without the earplug, with the dependent variables measured during the monaural listening condition. The rationale was to observe whether the effect of the plug (which could vary as a function of ear canal shapes) could modify the performance and the feelings experienced during monaural listening. However, we did not find any significant effects (all *ps* > 0.06, without correcting for multiple comparison).

Note that in this study we consistently plugged the left ear of participants to alter their hearing experience. Previous observations (Valzolgher et al., 2020a; Strelnikov et al. 2011; Mendonça, 2014) suggested that this could lead to different effects on sound localization toward the ipsilateral (left) or contralateral (right) side of the plug. Thus, to further deepen our analysis, we conducted the same analysis presented in Sect. "Reaching vs. naming sounds in binaural and monaural listening" of the manuscript on absolute errors, signed errors, confidence, and sound duration, while incorporating another independent variable: the side of space (right, left). The analysis, entirely reported in the Supplementary Materials, confirmed previously documented effects of plugging an ear on sound localization as a function of side, but they did not contribute to explaining the differences linked to aspects of confidence between reaching and naming, which were the main objectives of the study. For this reason, they will not be discussed further.

## Discussion

In the present study, we investigated if confidence in the perceived position of a sound changes depending on whether the interaction with the sound source entails planning and executing a sound-directed action or not. To this aim, we engaged participants in a sound localization task and asked them to rate, after each trial, their confidence in the perceived position of the sound. Crucially, they interacted with sounds sources either by reaching them or by naming the corresponding label, either in binaural or simulated monaural listening. First, we found that plugging one ear increased errors, reduced confidence and increased effort. While changes in sound localization accuracy in simulated monaural listening are well-documented in the literature, changes in confidence were described only in one previous work (Rabini et al., 2020) and the experience of listening effort remained largely overlooked with respect to sound localization. Second, we observed that sound localization accuracy did not differ between reaching and naming conditions, and yet participants felt more confident and needed less time to respond to the sound when reaching to the sources compared to naming them, regardless of the listening condition. These results expand the literature on the role of motor responses on perceived confidence (Sanchez et al., 2023; Siedlecka et al., 2021; Wokke et al., 2020) to the domain of hearing. Third, using a novel index of metacognitive processing (i.e., the internal coherence index), we found that participants found it harder to maintain a coherent criterion between performance and confidence when temporarily exposed to monaural compared to binaural listening.

The first strength of this study lies in its consideration of uncertainty as a dependent variable in the context of sound localization. As mentioned in the introduction, the analysis of acoustic space is often accompanied by uncertainty. However, studies on spatial hearing have almost entirely focused on measures of accuracy and precision, neglecting the subjective uncertainty associated with the perceived sound location. In acoustic perception and, particularly, in speech-in-noise research, other studies have focused on subjective listening experience beyond performance. In particular, many studies have examined listening effort (Peelle, 2018; Pichora-Fuller et al., 2016, Van Den Tillaart-Haverkate et al., 2017) and relatively less work has examined confidence associated with speech processing (e.g., Giovanelli et al., 2021; Hakkani-Tur et al., 2005). However, in the domain of spatial hearing, these investigations remain limited. Two notable exceptions are the study by Rabini et al. 2020 mentioned in the introduction, as well as the recent study from our group (Valzolgher et al., 2024), which introduced direct and indirect measures of uncertainty in a sound localization task within a noisy context. Valzolgher et al. (2024) asked participants to manipulate the dimensions of a sphere representing the sound source's position until they were confident that it included the source's location. The authors described the experience beyond performance and documented that reported uncertainty (as well as the size of the sphere) increased with higher levels of background noise.

In the present study, we measured confidence both directly, by asking participants to rate their confidence using a Likert scale, and indirectly, by using sound duration as an index of uncertainty. Using timing (e.g. reaction time) as an index to describe the experience beyond performance has been done by other authors studying listening abilities, especially as an indirect measure of listening effort (Houben et al., 2013). Here, we investigated this temporal variable in relation to the feeling of uncertainty and documented a relationship between these two variables. However, we believe that these pioneering examples (including our study) represent only a first step toward conceptualizing the experience of acoustic space as something that extends beyond performance. This is particularly relevant considering the listening experience of individuals with hearing deficits. Taking these variables into account could help to describe the listening experience in its totality as the feelings people experienced during a task often determine the way they behave in a given situation: solving it or avoiding it (see for instance Inzlicht et al., 2021).

### Reaching to sounds lead to perceive more confidence in one’s response on the sound’s position

The main finding of our experimental study is that reaching to sounds leads to perceiving more confidence in one’s response on the sound’s position. This corroborates and expands previous evidence about the role of motor planning in confidence judgments (Sanchez et al., 2023; Siedlecka et al., 2021; Wokke et al., 2020). This result is in line with the notion that perceived confidence combines both the strength of the incoming sensory signals and the decision-making processes that occur when people are asked to provide a response (Kiani & Shadlen, 2009). Considering our study, the process of weighting both uncertainty signals could occur during both types of response, reaching or naming. However, what distinguishes reaching to sounds from naming is the involvement of mechanisms and circuits linked to the implementation of motor actions evoked by incoming sensory signals. Programming a motor action toward a specific position in space may have an intrinsic component of uncertainty, linked to the motor execution of the gesture itself. This uncertainty must be weighed with the other components relating with sensory signals and decision-making. Additional information provided by motor actions to the uncertainty estimation relates to how people evaluate its success with relation to the goal (i.e., if they think the controller reach the correct sound source). This evaluation is the result of two components. The first one is a prospective component, referring to the information available before action execution. It concerns, for instance, the representation of the planned movement at a higher level (Fleming et al., 2015; Sanchez et al., 2023), which is also influenced by prior knowledge about stimuli and environment (Fassold et al., 2023). As suggested by Fleming and colleagues (2015), higher-level action representations in dorsal premotor cortex, rather than lower-level activity in the primary motor cortex, contribute to subjective confidence. Confidence estimation is not a matter of doing a movement, but it is the decision to implement an action triggered by a certain object. The second component is the retrospective one, referring to the information available after-action execution, e.g. the feedback provided by the proprioceptive information about body position in space (Fassold et al., 2023).

By documenting an increase in confidence rating when reaching toward sounds vs. naming the labels, our study corroborates the idea that in formulating confidence judgments, people consider a whole series of information that goes beyond the (first order) analyses of sensory inputs, and comprise further information as internal cues related to decision making and action planning (Fleming 2021; Kool et al., 2010).

### Further elements of the relationship between motor action and auditory spatial confidence

Another aspect that differs between naming and reaching is that reaching toward a sound source to stop the sound can give to the actor a higher sense of agency. The sense of agency, i.e., the feeling of causing or generating actions or events in the external world (Gallagher, 2012) may also play a role in the assessment of one's confidence in sensorial perception. Acting on an object could bring a higher sense of agency, especially in our experiment, where the sound stopped when participants acted on the stimulus. The chance to stop the sound should bring a higher sense of agency, translating into a greater feeling of control over the external world. This could increase confidence in their perceptual sensations and subsequent choices. In our study, we attempted to control the feeling of agency by having participants' responses stop the sound in both conditions. However, our study has the limitation of not thoroughly investigating the feeling of agency experienced during the task. We only have a few anecdotal reports of some participants feeling a greater sense of agency when reaching. Future studies are needed to explore the effect of the feeling of agency on confidence more deeply. For instance, they could manipulate the sense of agency more systematically (e.g., by comparing a high-agency condition to a low-agency condition) and by asking participants to directly rate their perceived sense of agency.

This hypothesis is coherent with the Self-Determination Theory (Ryan & Deci, 2000), that posit an advantage of the paradigm with a physical response due to the increased engagement and motivation that the listener experiences while performing the task. This increased engagement may also have been experienced by participants involved in the reaching interaction. Future studies should explicitly measure engagement and motivation of participants, as they could contribute to explaining the difference in performance between the two different response conditions.

Attention may also have played a role in the higher confidence ratings in the reaching vs. naming condition. Reaching toward sounds requires the coordination of different effectors, such as the hand and the eyes, within a common reference frame (Cohen & Andersen, 2002). In turn, this could enhance attention orienting and potentially result in a more stable (or salient) spatial coding of the sound source location (see Valzolgher et al., 2020a for discussion).

As anticipated in the introduction, although visual objects usually evoke actions toward them, it is also possible to perform actions toward acoustic objects. Motor actions toward acoustic objects could aim at reaching them, as in the case of searching for a ringing telephone, or at manipulating them, as when turning the knob to adjust the volume of a radio. However, the actions that can be implemented toward acoustic objects do not only involve the hand, but the entire body. An interesting example is the case of blind football in which players use auditory cues to reach, control and hit the ball (emitting sound when it moves) using their feet. These players have developed a particular skill in localizing sounds (Mieda et al., 2019; Velten et al., 2014) also thanks to the fact that this sport exploits the relationship between motor and coordination skills and the ability to perceive the position of the sound in space. However, imagining more common activities, it is possible to think of actions toward sound objects that involve the orientation of the head toward the sound, which plays a crucial role in its localization (Kato et al., 2003; McAnally & Martin, 2014; Thurlow et al., 1967; Valzolgher, 2024; Wallach, 1940) or the orientation of the eyes toward the direction of the sound. In our study, we restricted head movement for experimental purposes (see Methods section) and did not track eye movements due to the absence of an integrated eye-tracker in the headset. Nevertheless, we hypothesize that in our experiment, eye movements were likely similar in both experimental conditions. In one scenario, participants would have needed to direct their gaze to execute a reaching movement, while in the other, they would have needed to do so to read labels. If a disparity were to exist between the two conditions, it is probable that participants could execute reaching movements even in peripheral vision, a likelihood that diminishes in the naming condition. Thus, our study implies that the execution of reaching actions involves processes beyond mere orientation of gaze toward a spatial direction, although further investigations involving eye movements could enhance the depth and validation of our hypotheses. These considerations highlight an additional aspect worth exploring in future studies. While our research showed that confidence increases in reaching tasks, the role of information from different sensory modalities in shaping these confidence judgments remains unclear. Specifically, it is yet to be determined whether multisensory inputs, such as visual information, influence confidence judgments related to a single sensory modality, like audition. For instance, could the ability to see sound sources enhance confidence in judgments based on acoustic spatial cues? To the best of our knowledge, no studies have directly addressed this question so far. However, recent work by Maddox and collaborators has demonstrated advantages of visual input on spatial hearing (Atilgan et al., 2018; Maddox et al., 2015), including those arising from auditory-oculomotor interactions (Maddox et al., 2014). For instance, these authors have shown that the presence of visual stimuli associated with auditory stimuli improves performance in auditory tasks, regardless of the informativeness of the visual cues. This type of advantage cannot be explained by a Bayesian causal inference model, which assumes a late integration of multisensory cues (Cappelloni et al., 2019). The authors have attempted to formulate a series of alternative models to explain this phenomenon, such as the possibility of earlier integration or bottom-up attentional models. Extending the investigation to the more metacognitive aspects of this experience could help further explore this phenomenon. Although these reflections remain speculative, they highlight potential future directions for investigating multisensory integration in confidence judgments.

### Toward a metacognition of spatial hearing

This research has demonstrated the utility of studying confidence in perceptual experiences, particularly in the context of sound localization. It also indicates that there may be modulations of this subjective perception based on whether or not one acts on the sound. However, studying confidence is only an initial step toward a more comprehensive exploration of metacognition in spatial hearing. Metacognition is a cognitive function that allows us to reflect on our own thinking processes, permitting for the monitoring and control of cognitive performance and processes (Efklides, 2008; Flavell, 1979). To simplify, having good metacognitive monitoring means being able to associate confidence judgments consistently with one's performance. In other words, it refers to the feeling of being confident about performance when it is correct and unconfident when performance decreases (Fleming et al. 2024). While metacognition in listening has been partially explored (e.g., Giovanelli et al., 2021; Hakkani-Tur et al., 2005), metacognition in spatial hearing remains largely uncharted. However, delving into this area would be both intriguing and beneficial, as there are numerous scenarios where individuals may struggle to locate sound sources, and it is uncertain whether they are aware of their limitations. Our study takes an initial step in this direction by examining the trial-by-trial consistency between confidence and performance. Interestingly, we found a difference between the binaural and simulated monaural listening conditions. This finding is consistent with data of a previous study conducted by Rabini et al. (2019). Even if the authors did not explicitly use the term “metacognition”, they still attempted to combine performance and confidence. In our study, when listening with one ear plugged, people had more difficulty in making confidence judgments consistent with performance as compared to binaural condition. Similarly, in Rabini et al. (2020) during monaural listening there was a lower percentage of trials in which high performance corresponded to high confidence and low performance to low confidence compared to the binaural listening situation. Taken together, these results may suggest a difficulty in adjusting confidence judgments when the perceptual experience deviates from our everyday experience. It is possible that it takes time to understand one's own way of behaving in a new listening context and therefore to judge one's confidence coherently with performance. This point suggests a more general implication that operating in familiar vs. novel situations may allow better cognitive estimates of our abilities. As this awareness plays a crucial role in learning mechanisms (Dehaene, 2020), this consideration may be of relevance also for rehabilitation practices.

Moreover, in our study, we did not show differences between the reaching and the naming condition by considering this index. A possible explanation is linked to what was suggested in the previous paragraph. Also in this case, to observe effects on metacognition it might expect a slower process of adaptation to the type of task. Considering changing of this capacity over time paves the way for future studies. They might explore the relationship between action and confidence (and metacognition) by adopting longitudinal experimental designs. The development over time of the ability to make confidence judgments consistent with one's own performance could be particularly interesting for the study of relearning and could be crucial for developing training paradigms aimed at using internal feedback as a key to know one's own ability and consequently finding ways to improve it (Hewitson et al., 2023). These future studies could also be particularly relevant from a clinical point of view. Localizing sound sources is often accompanied by uncertainty about sound position and this is particularly true if considering people with hearing deficits, who struggle to localize sound sources due to alterations in auditory cues. Spatial hearing for these individuals is a challenging experience, as they are constantly uncertain about sounds positions’ and need to rely on other modalities (Zheng et al., 2022). Learning to use one's own confidence as a “feedback tool” could lead people with acoustic impairment to implement effective strategies (see Nicol, 2021; Meyniel et al., 2015; Zonooz et al., 2018) and, for this reason, training metacognition could prove to be particularly impactful in their lives.

The extent to which ‘confidence’ serves as an indicator of learning remains to be investigated. On one hand, a progressive increase in confidence (linked to good metacognition) could suggest improvement in the learning process. On the other hand, if this increase is associated with an overestimation of one’s abilities, it may lead to a stagnation in the acquisition of new skills or the implementation of new strategies. It is worth noting that here we refer to an explicit confidence judgment, though more implicit aspects, such as the uncertainty associated with perceptions (see Parr & Friston, 2017; Deroy et al., 2016), may also play a role.

## Supplementary Information

Below is the link to the electronic supplementary material.

Supplementary file1 (DOCX 27 KB)

## Data Availability

The datasets generated and analysed during the current study are available in OSF repository, and retrieved from osf.io/rsvhp.
