# Supplementary material for: Action toward sound sources enhances auditory spatial confidence: on the metacognitive consequences of reaching to sounds
Source: Psychol Res. 2025 Jan 19;89(1):48. doi: 10.1007/s00426-025-02079-3 (PMC11743395; doi:10.1007/s00426-025-02079-3)
Supplement: Supplementary file 1 — Supplementary file1 (DOCX 27 KB) [file 426_2025_2079_MOESM1_ESM.docx]

**Action toward sound sources enhances auditory spatial confidence:**

**on the metacognitive consequences of reaching to sounds**

Chiara Valzolgher^1^*, Lisa Lever^1,2^*, Tommaso Rosi^4^, & Francesco Pavani^1,2,3^

1. Center for Mind/Brain Sciences (CIMeC), University of Trento, Italy

2. Department of Psychology and Cognitive Sciences (DiPSCo), University of Trento, Italy

3. Centro Interuniversitario di Ricerca "Cognizione, Linguaggio e Sordità" (CIRCLeS), Trento, Italy

4. Level UP, Trento, Italy

* These authors equally contributed to the present work.

**SUPPLEMENTARY MATERIALS**

**The role of side**

To further deepen our analysis, we conducted the same analysis presented in section 3.1 of the main manuscript on absolute errors, signed errors, confidence, and sound duration, while incorporating another independent variable: the side of space (right, left). This was done because we consistently plugged the left ear, and previous observations (Valzolgher et al., 2020; Strelnikov et al., 2011; Mendonça, 2014) suggest that this could lead to different effects on sound localization towards the ipsilateral (left) or contralateral (right) side of the plug. Here, we report only the main effect and the interaction involving side, as the other effects have already been reported in section 3.1.

ANOVA on absolute error revealed a main effect of side (*F*(1,27)= 5.81, *p* = 0.02, *ŋ^2^* = 0.03) and an interaction between side and listening condition (*F*(1,27)= 8.92, *p* = 0.006, *ŋ^2^* = 0.05) reflecting the fact that during monaural listening absolute error was higher when the sound were emitted from the plugged (left) side of the space (binaural: left: 1.35±0.93; right: 2.30±1.44, *t* =-0.46 , *p* =1.00; monaural: left: 19.29±13.65; right: 11.30±7.71, *t* =3.82, *p* = 0.002). Plus, we observed an interaction between side and response type (*F*(1,27)= 6.05, *p* = 0.02, *ŋ^2^* = 0.002) revealing that left and right side differed during the reaching (*t* =2.90, *p* =0.04), but not during the naming (*t* =1.79, *p* =0.50). We attributed this effect of the use of right effector during reaching condition which may led to smaller error in the right and higher effort in the left part of the space.

ANOVA on signed error revealed a main effect of side (*F*(1,27)= 42.22, *p* < 0.001, *ŋ^2^* = 0.20) and an interaction between side and listening condition (*F*(1,27)= 55.03, *p* < 0.001, *ŋ^2^* = 0.23) reflecting the fact that during monaural listening signed error was more positive (i.e. bias toward right) when the sound were emitted from the plugged (left) side of the space (binaural: left: -0.09±1.50; right: 0.69±2.33, *t* =-0.36 , *p* =1.00 ; monaural left: 14.33±17.48; right: -6.91±10.88, *t* =9.82, *p* <0.001).

ANOVA on confidence rating revealed a main effect of side (*F*(1,27)= 38.46, *p* < 0.001, *ŋ^2^* = 0.05) and an interaction between side and listening condition (*F*(1,27)= 52.91, *p* < 0.001, *ŋ^2^* = 0.07) reflecting the fact that during monaural listening confidence decreased was lower when the sound were emitted from the plugged (left) side of the space (binaural: left: 4.80±0.52; right: 4.76±0.51, *t* =0.47, *p* =1.00 ; monaural left: 3.20±0.95; right: 3.98±0.81 , *t* =-9.50, *p* <0.001).

ANOVA on sound duration revealed a main effect of side (*F*(1,27)=29.89, *p* < 0.001, *ŋ^2^* = 0.06) and an interaction between side and listening condition (*F*(1,27)=16.99, *p* < 0.001, *ŋ^2^* = 0.04) reflecting the fact that during monaural listening sound duration was longer when the sound were emitted from the plugged (left) side of the space (binaural: left: 3.86±1.32; right: 3.80±1.26, *t*=0.50, *p*=1.00; monaural left: 5,28±1.96; right 4.40±1.55, *t*=6.70, *p*<0.001). Plus, we observed an interaction between side and response type (*F*(1,27)=5.60, *p*=0.03, *ŋ^2^*=0.004) revealing that a small (even if not significant if corrected with Bonferroni) difference between reaching and naming emerged when sounds were emitted by the right speakers (right reaching: 3.90±1.45; right naming: 4.30±1.38; *t*=2.50, *p*=0.10), but not the left ones (left reaching: 4.50±1.44; left naming: 4.64±1.63; *t*=0.89, *p*=1.00). Particularly, for the right side, the sounds were longer when participants named the labels as compared to when they reached them. Again, this effect is probably due to the use of right hand which is closer to the right side of the space.

In summary, while these analyses were useful in confirming the previously documented effects of plugging an ear on sound localization as a function of side, they did not contribute to explaining the differences linked to aspects of confidence between reaching and naming, which were the main objectives of the study. For this reason, they will not be discussed further.

**Supplementary Figure 1**

**
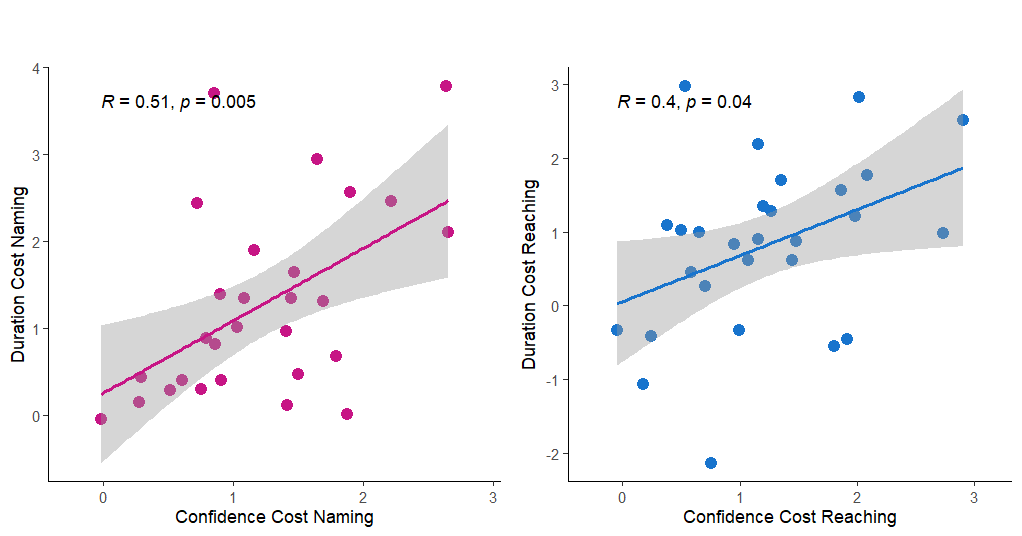
**

Pearson correlation between confidence cost of changing the listening condition from monaural to binaural (computed as the difference in confidence between listening conditions, for each participant) and the sound duration cost for the same change in listening condition (computed as the difference in sound duration between listening conditions, for each participant) separately for naming (A) and reaching (B).
